# NUCB2/nesfatin-1 Is Associated with Elevated Levels of Anxiety in Anorexia Nervosa

**DOI:** 10.1371/journal.pone.0132058

**Published:** 2015-07-10

**Authors:** Tobias Hofmann, Anne Ahnis, Ulf Elbelt, Matthias Rose, Burghard F. Klapp, Andreas Stengel

**Affiliations:** 1 Charité Center for Internal Medicine and Dermatology, Division for General Internal and Psychosomatic Medicine; Charité, Universitätsmedizin Berlin, Berlin, Germany; 2 Charité Center for Internal Medicine with Gastroenterology and Nephrology, Division for Endocrinology, Diabetes and Nutrition, Charité, Universitätsmedizin Berlin, Berlin, Germany; Sapienza University of Rome, ITALY

## Abstract

**Objective:**

NUCB2/nesfatin-1 is an anorexigenic hormone with elevated levels in obese and decreased levels in anorexia nervosa (AN) patients. Moreover, a role in the regulation of stress and emotions was suggested by several rodent and preliminary human studies. Since anxiety and depression are common comorbidities in AN, we investigated the association of NUCB2/nesfatin-1 with anxiety, depression and perceived stress in AN.

**Methods:**

We analyzed circulating NUCB2/nesfatin-1 levels in 64 female inpatients diagnosed with anorexia nervosa (body mass index, BMI; mean±SD, 14.7±2.3 kg/m^2^). At the same time anxiety (GAD-7), depression (PHQ-9), stress (PSQ-20) and disordered eating (EDI-2) were measured psychometrically.

**Results:**

No correlation was observed between NUCB2/nesfatin-1 and BMI (*r* = 0.06, *p* = 0.70). The study population was divided in patients with low anxiety (n = 32, GAD-7 scores, mean±SD, 7.5±3.3) and high anxiety (n = 32, 16.0±3.0, *p*<0.001). Patients with high anxiety scores displayed 65% higher NUCB2/nesfatin-1 levels (*p* = 0.04). This was reflected by a positive correlation of GAD-7 and NUCB2/nesfatin-1-levels (*r* = 0.32, *p* = 0.04). Scores of PSQ-20 (73.3±14.3 *vs*. 48.6±17.2) and PHQ-9 (18.8±5.0 *vs*. 10.3±5.1) were higher in the high anxiety group (*p*<0.001) but did not correlate with NUCB2/nesfatin-1 (*p*>0.05). EDI-2 total score was also higher in the high anxiety group (52.3±14.1 *vs*. 40.2±16.0, *p* = 0.02), while no correlations of EDI-2-scores with plasma NUCB2/nesfatin-1 were observed (*p*>0.05).

**Conclusions:**

Circulating NUCB2/nesfatin-1 levels correlated positively with perceived anxiety, whereas no association with BMI or eating disorder symptoms was observed. NUCB2/nesfatin-1 might be primarily involved in the modulation of anxiety and subsequently in the regulation of eating habits and body weight in AN.

## Introduction

Nesfatin-1 is an 82-amino-acid peptide hormone cleaved from nucleobindin2 (NUCB2). First described in 2006 [1], nesfatin-1 has been considered to be primarily involved in the regulation of hunger and satiety as an anorexigenic modulator since intracerebroventricular and also peripheral injection were shown to reduce food intake in rodents [1, 2]. NUCB2/nesfatin-1 is expressed in several cerebral regions of the rat such as hypothalamus and brainstem [1], amygdala [3–5], and parasympathetic and sympathetic neurons [5]. In humans, it was also detected in the Edinger-Westphal nucleus [6]. In addition, NUCB2/nesfatin-1 has been shown to occur also peripherally in human and murine adipose tissue [7]. It is colocalized with insulin in rodent [8] and human pancreatic beta-cells [9] and colocalized with ghrelin in gastric human P/D_1_- and rat X/A-like cells [10, 11]. Interestingly, the stomach seems to be the major source of NUCB2/nesfatin-1 as expression levels are higher compared to the brain as shown in rats [10]. Since most studies did not distinguish between full length NUCB2 and processed nesfatin-1 due to the fact that the antibodies used also recognize full length NUCB2 (for discussion see [12]) the analyte should be referred to as NUCB2/nesfatin-1.

The regulation of circulating NUCB2/nesfatin-1 seems to depend on food ingestion with decreased levels after fasting and a restoration after re-feeding in rats [10], while confirmation of these data in humans is pending. In addition to the short term alterations, also sustained changes in body weight have been reported to affect NUCB2/nesfatin-1. The only study conducted in subjects with anorexia nervosa reported lower NUCB2/nesfatin-1 levels compared to healthy controls [13]. In line with these data several studies showed a positive correlation of NUCB2/nesfatin-1 levels with body mass index (BMI) [7, 14]. However, this association is not yet fully established as other studies reported an inverse relationship [15–18] or no significant association of NUCB2/nesfatin-1 levels and BMI [19]. Alternatively, other factors might more strongly influence NUCB2/nesfatin-1 levels and thereby alter the expected relationship between NUCB2/nesfatin-1 and BMI [19].

Recently, NUCB2/nesfatin-1 has been suggested to be also a part of circuitries involved in the modification of behavioral and emotional responses to stress [20]. In rodents, intracerebroventricular injection of nesfatin-1 – besides reducing food intake – induced anxiety-related behaviors [21]. Subsequently, several groups showed an activation of NUCB2/nesfatin-1 neurons in rats undergoing restraint stress, a well-established rodent model of psychological stress [22–24]. In humans, elevated NUCB2 mRNA expression was observed in the brain Edinger-Westphal nucleus [6] and higher NUCB2/nesfatin-1 peptide levels in the peripheral circulation [25] of depressed patients compared to healthy controls. In line with these findings, a positive correlation of anxiety, depression and perceived stress with circulating NUCB2/nesfatin-1 levels has been reported in obese women [19]. Interestingly, male patients with general anxiety disorders displayed decreased nesfatin-1 levels [26] pointing towards a possible sex-specific regulation of NUCB2/nesfatin-1.

The involvement of NUCB2/nesfatin-1 in the regulation of food intake and emotion should be viewed in an integrated manner since mood and eating habits seem to be interrelated. Moreover, a hormone with anorexigenic and anxiogenic effects might play a role in the etiology or maintenance of anorexia nervosa which is often accompanied by depression and anxiety [27–30]. In the present study, we therefore investigated the relationship between NUCB2/nesfatin-1 and different psychological parameters and hypothesized a positive correlation of NUCB2/nesfatin-1 primarily with anxiety which has been shown by our group in obese women before [19]. We also expected positive associations of NUCB2/nesfatin-1 with depression and perceived stress as also shown for obese women in the same study [19]. In addition, we tested whether patients with high anxiety also display higher scores of depression and perceived stress and exploratively investigated whether any associations with symptoms of disordered eating could be observed. Lastly, in a small sample we compared circulating NUCB2/nesfatin-1 levels of anorexia nervosa patients with normal weight patients matched for sex, age and anxiety scores.

## Materials and Methods

### Ethics statement

All investigations were conducted according to the Declaration of Helsinki and all patients gave written informed consent. The study was approved by the institutional ethics committee of the Charité – Universitätsmedizin Berlin (protocol number: EA1/114/10).

### Subjects

We enrolled 64 female inpatients suffering from anorexia nervosa (BMI 14.7 ± 2.3 kg/m^2^; range 8.7–19.3 kg/m^2^) and 10 normal weight female inpatients (BMI 20.5 ± 1.6 kg/m^2^; range 18.6–23.4 kg/m^2^) at admission to their treatment (consisting of biomedical therapy and physiotherapy as well as both individual and group psychotherapy, music and art therapy and body psychotherapy) in the Division of General Internal and Psychosomatic Medicine at Charité –Universitätsmedizin Berlin. Inclusion criteria for anorexia nervosa patients encompassed female sex and the fulfillment of ICD-10 (International Statistical Classification of Diseases and Related Health Problems of the World Health Organization, 10^th^ revision) diagnostic criteria for anorexia nervosa (typical and atypical anorexia nervosa as well as restricting and binge-purge subtypes) [31]. The atypical subtype was defined as meeting all but one criterion of the typical form (BMI below 17.5 kg/m^2^, self-induced weight loss, body image distortion, and amenorrhea) [31]. ICD-10 criteria for anorexia nervosa basically equal DSM-IV (Diagnostic and Statistical Manual of Mental Disorders, Fourth Edition) criteria [32], while in contrast to the DSM-V (Diagnostic and Statistical Manual of Mental Disorders, Fifth Edition) criteria [33] amenorrhea is still a criterion for typical anorexia nervosa. Inclusion criteria for normal weight patients comprised a BMI of 18.5 to 25.0 kg/m^2^, female sex and the absence of relevant biomedical diagnoses. These patients were admitted to the hospital for the treatment of somatoform, anxiety, depressive or adjustment disorders. Patients with somatoform disorders of the gastrointestinal system were excluded. Exclusion criteria for the whole study were current pregnancy, psychotic disorders and an age below 18 years.

### Anthropometric measurements

Body weight and height were assessed at the same day of blood withdrawal between 07:00–08:00 am in light underwear and BMI was calculated as kg/m^2^.

### Laboratory analyses

Venous blood samples were taken after an overnight fast between 7:00 and 8:00 in the morning. Patients were allowed to drink small amounts of water but were advised not to drink larger amounts or other beverages, to eat, smoke or exercise before blood withdrawal. All blood samples were collected within 3 days after admission to avoid biases due to the onset of metabolic changes following treatment initiation. The blood was collected in pre-cooled standard EDTA tubes prepared with aprotinin (1.2 Trypsin Inhibitory Unit per 1 ml blood; ICN Pharmaceuticals, Costa Mesa, CA, USA) for peptidase inhibition. Immediately after blood withdrawal the tubes were stored on ice and then centrifuged at 4°C for 10 min at 3000 g. Plasma was then separated and stored at -80°C until further processing. NUCB2/nesfatin-1 plasma levels were measured using a commercial enzyme-linked immunosorbent assay (ELISA, catalog # EK-003-26, Phoenix Pharmaceuticals, Inc., Burlingame, CA, USA). All samples were processed at once (intra-assay variability was 5%, inter-assay variability was 7%). The antibody used in this ELISA was raised against nesfatin-1 and also recognizes full length NUCB2 containing the epitope.

### Psychometric questionnaires

For psychometrical examination and *ad hoc* questions about socioeconomic status patients were given personal digital assistants (PDA) one or two days before or on the day of blood withdrawal and asked to fill in the following questionnaires within one day.

For assessment of anxiety and depression two scales of the self-report measure patient health questionnaire (PHQ) [34] were used. For the diagnosis of a generalized anxiety disorder specificity was 0.92 and sensitivity 0.89 [35]. According to the English version, the German version of the GAD-7 [36] utilizes 7 items designed to diagnose general anxiety disorder [35] but also captures panic, social anxiety or posttraumatic stress symptoms. Cronbach’s alpha for the present sample was 0.83.

The PHQ-9 depression scale [34], consisting of 9 items, is a widely used screening instrument for determination of the severity of depressive symptoms. We administered the German version by Löwe et al. [37]. For the current population Cronbach’s alpha was calculated as 0.86. In a meta-analysis of 17 validation studies in different languages including the German language translation, specificity was 0.92 and sensitivity 0.80 for the diagnosis of a major depressive disorder [38].

For the measurement of stress we used the perceived stress questionnaire (PSQ) [39] in its revised German version with 20 items (PSQ-20) [40]. The PSQ emphasizes the subjective perception of stress and additionally provides four subscales evaluating “worries”, “tension”, and “joy” as stress responses and “demands” as a perception of external stressors. Cronbach’s alpha for the subscales ranged from 0.83 to 0.87.

The Eating Disorder Inventory (EDI) was developed [41] to assess eating disorder symptoms in anorexic and bulimic patients and is also a self-report instrument encompassing 64 items on 8 subscales measuring “drive for thinness”, “bulimia”, “body dissatisfaction”, “ineffectiveness”, “perfectionism”, “interpersonal distrust”, “interoceptive awareness”, and “maturity fears”. We used the German version [42] of the 2^nd^ version [43] which added 3 subscales to the original one but only used the above mentioned first 8 subscales of the EDI-2. Cronbach’s alpha was determined as 0.87.

### Statistical analysis

Distribution of the data was determined by Kolmogorov-Smirnov test. For descriptive characterization of demographic and anthropometric data, t-tests and Mann-Whitney-U-tests were employed depending on the distribution of the data. For descriptive characterization of socioeconomic data and anorexia nervosa subtypes frequencies were calculated using crosstabs followed by χ^2^-tests. Correlations were determined by Pearson’s or Spearman’s analyses depending on the distribution of the data. Differences in outcome variables between groups were calculated using t-tests. Differences between groups were considered significant when *p* < 0.05. Data are expressed as mean ± standard deviation (SD) for parametric data and as median (25^th^ percentile/75^th^ percentile) for non-parametric data. Statistical analyses were conducted using SigmaStat 3.1 (Systat Software, San Jose, CA, USA).

## Results

According to the results in the GAD-7 we divided our sample into two groups, one with low and one with high anxiety levels by splitting the sample at the median score of 12.

Demographic and socioeconomic characteristics, duration of disease and subtypes of the anorexia nervosa study population (n = 64) are described in Table 1. Comorbidities and additional laboratory analyses in anorexia nervosa patients are shown in S1 Table.

**Table 1:** Demographic and socioeconomic characteristics, duration of disease and subtypes of anorexia nervosa of the anorexia nervosa study population.

Parameter	Low anxiety (n = 32)	High anxiety (n = 32)	Missing data	*p*
*Demographic characteristics*				
Age (years)	23.0 (19.0/30.5)	25.0 (21.3/31.3)	0	0.33^a^
Body mass index (kg/m^2^)	14.8 ± 2.0	14.6 ± 2.6	0	0.73^b^
*Duration of disease*	6.0 (1.0/14.0)	6.0 (1.1/11.2)	1	0.94^a^
*Socioeconomic characteristics*				
Living in a partnership (yes/no)	11/17	9/20	7	0.51^c^
Level of education			7	0.88^c^
university entrance diploma	7	11		
vocational diploma	2	2		
level 1 certificate	14	12		
certificate of secondary education	4	3		
without	1	1		
Current employment (yes/no)	7/21	7/22	7	0.94^c^
Unemployment during past 5 years (yes/no)	10/18	10/19	7	0.92^c^
*Anorexia nervosa subtype*			0	0.37^c^
restricting type	11	16		
purging type	10	6		
atypical anorexia nervosa	11	10		

Statistical analyses: Normal distribution was determined by Kolmogorov-Smirnov test. Differences between groups:

^a^ Mann-Whitney-U-test, data expressed as median (25^th^ percentile/75^th^ percentile)

^b^ t-test, data expressed as mean ± standard deviation

^c^ χ^2^-tests.

### Circulating NUCB2/nesfatin-1 levels show a positive correlation with anxiety while they are not associated with depression and perceived stress in anorexic patients

The two study groups (low anxiety: n = 32, GAD-7 score range: 0 to 12, mean ± SD: 7.5 ± 3.3; high anxiety: n = 32, GAD-7 score range: 12 to 21, mean ± SD: 16.0 ± 3.0, *p* < 0.001) did not differ in age (*p* = 0.33; Table 1), or BMI (*p* = 0.79; Table 1).

**Fig 1:**
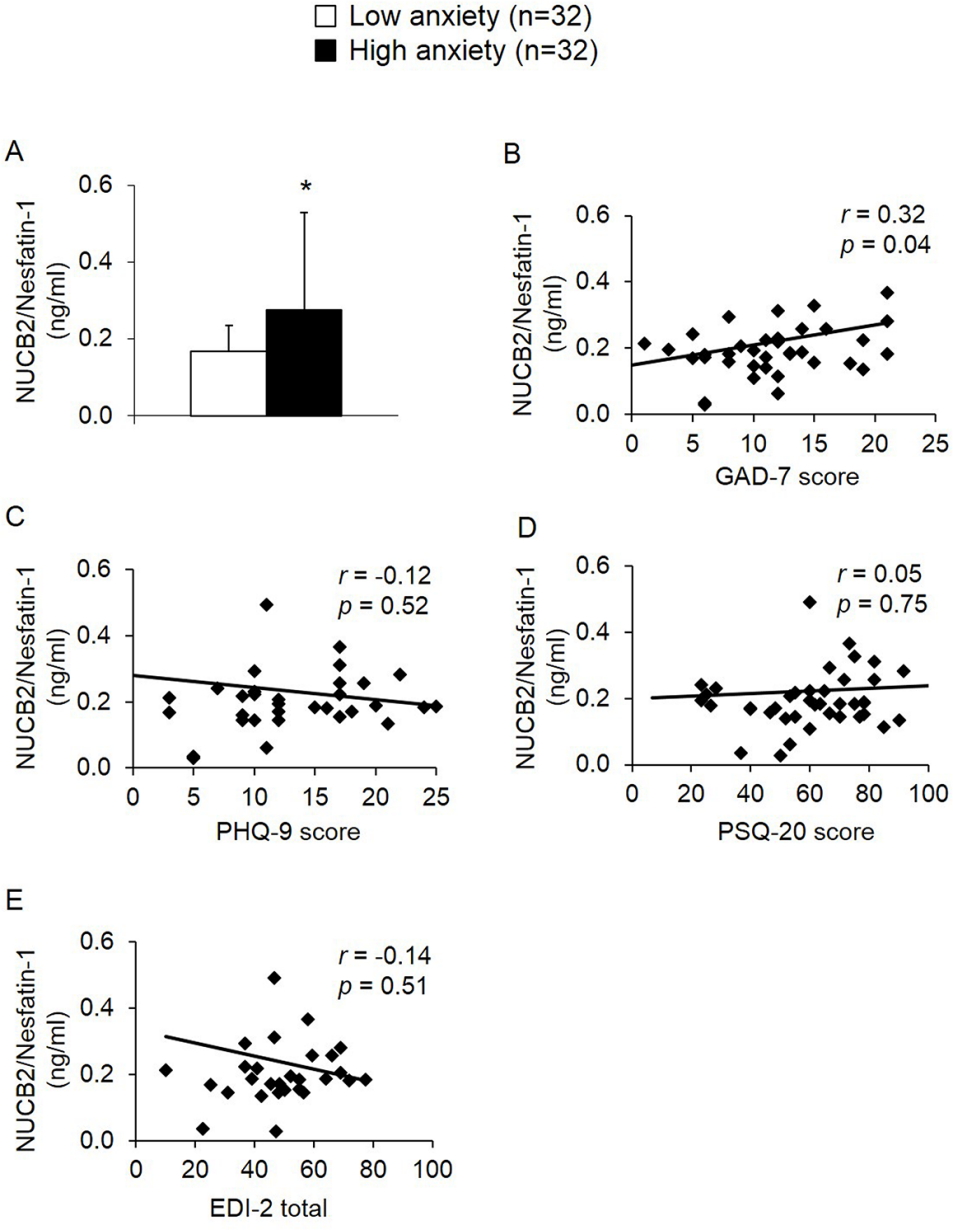
GAD-7, PHQ-9, PSQ-20 and EDI-2 scores and plasma NUCB2/nesfatin-1 levels in anorexic patients. Patients with high anxiety displayed significantly higher NUCB2/nesfatin-1 levels compared to those with low anxiety (A). This resulted in a positive correlation of NUCB2/nesfatin-1 with anxiety in the whole anorexic patient sample (B), whereas depression scores (C), perceived stress scores (D) and eating disorder symptoms (E) did not correlate with plasma NUCB2/nesfatin-1. Differences between groups were calculated using t-tests. Data are expressed as mean ± standard deviation. * *p* < 0.05. Distribution of the data was determined by Kolmogorov-Smirnov test. Correlations were determined by Pearson’s or Spearman’s analyses depending on the distribution of the data. Values for *r* and *p* are indicated in the graph. Abbreviations: EDI-2, eating disorder inventory; GAD-7, general anxiety disorder questionnaire; NUCB2, nucleobindin2; PHQ-9, patient health questionnaire depression; PSQ-20, perceived stress questionnaire.

The high anxiety group displayed 65% higher NUCB2/nesfatin-1 levels than the low anxiety group (0.28 ± 0.25 *vs*. 0.17 ± 0.07 ng/ml, *p* = 0.04; Fig 1A) which was reflected by a positive correlation of NUCB2/nesfatin-1 with GAD-7-scores in the whole study sample when treated as a continuous variable (*r* = 0.32, *p* = 0.04; Fig 1B). No correlations were observed for NUCB2/nesfatin-1 with age (*r* = 0.02, *p* = 0.89) or BMI (*r* = 0.06, *p* = 0.70; Table 2).

**Table 2:** Correlation of NUCB2/nesfatin-1 plasma levels with demographic and psychometric parameters of the anorexia nervosa study population (n = 64).

Parameter	*r*	*p*
Age	0.02	0.89
Body mass index (kg/m^2^)	0.06	0.70
GAD-7 score	**0.32**	**0.04**
PHQ-9 score	-0.12	0.52
PSQ-20 total score	0.05	0.75
worries	-0.09	0.61
tension	0.14	0.40
demands	-0.03	0.86
joy	-0.16	0.33
EDI-2 total score	-0.14	0.51
drive for thinness	-0.19	0.35
bulimia	-0.12	0.55
body dissatisfaction	-0.01	0.97
perfectionism	-0.22	0.28
interpersonal distrust	-0.02	0.94
ineffectiveness	-0.18	0.38
interoceptive awareness	0.05	0.80
maturity fears	-0.11	0.61

Statistical analysis: Normal distribution was determined by Kolmogorov-Smirnov test. Correlation analysis was conducted by Pearson’s or Spearman’s analysis, respectively. Significant correlations are displayed in bold. Abbreviations: EDI-2, eating disorder inventory; GAD-7, general anxiety disorder questionnaire; NUCB2, nucleobindin2; PHQ-9, patient health questionnaire depression; PSQ-20, perceived stress questionnaire.

**Fig 2:**
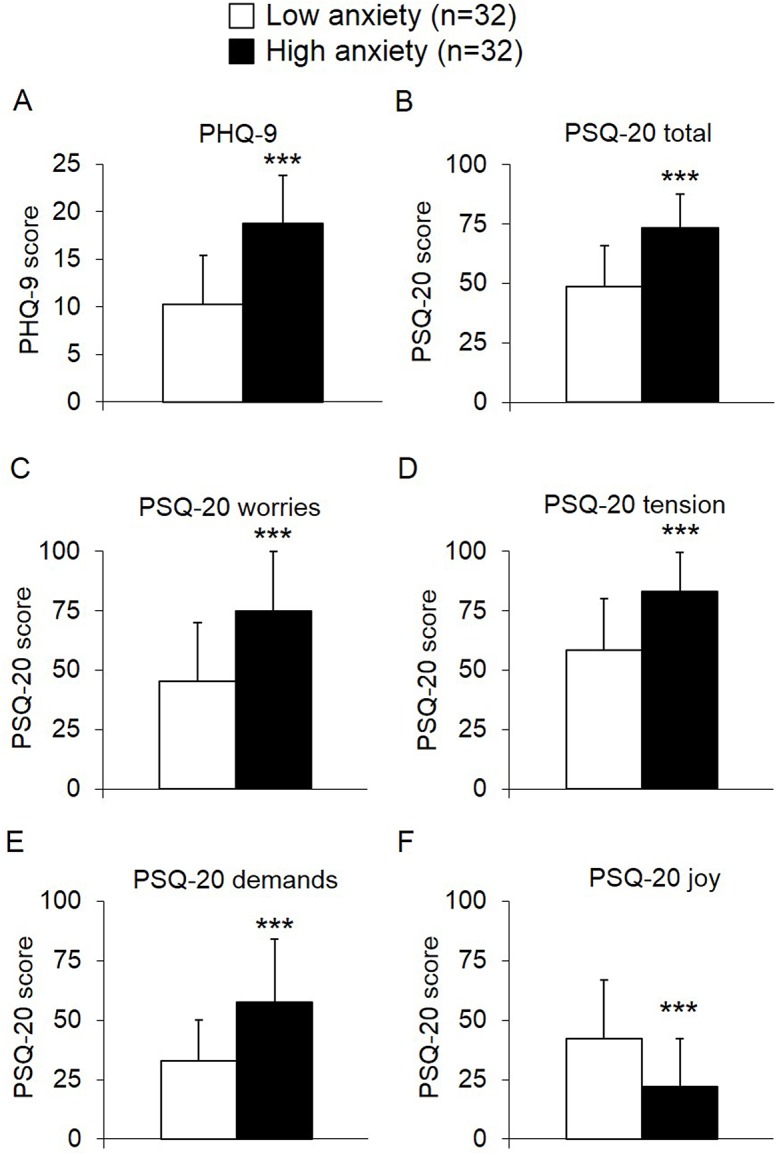
PHQ-9 and PSQ-20 scores of anorexic patients with low *versus* high anxiety. Patients with high anxiety scores displayed higher total scores in PHQ-9 (A) and PSQ-20 (B). Similarly, the PSQ-20 subscales differed between the two groups with higher scores for “worries” (C), “tension” (D) and “demands” (E) and lower scores for “joy” (F) in the high *versus* low anxiety group. Differences between groups were calculated using t-tests. Data are expressed as mean ± standard deviation. *** *p* < 0.001. Abbreviations: PHQ-9, patient health questionnaire; PSQ-20, perceived stress questionnaire.

As expected, the high anxiety group also showed a higher depression score (PHQ-9, +83%, *p* < 0.001; Fig 2A), perceived stress total score (PSQ-20, +51%, *p* < 0.001; Fig 2B) and higher scores on the PSQ-20 subscales “worries” (+66%, *p* < 0.001; Fig 2C), “tension” (+42%, *p* < 0.001; Fig 2D), and “demands” (+74%, *p* < 0.001; Fig 2E) and lower scores on the “joy” subscale (-47%, *p* < 0.001; Fig 2F) compared to the low anxiety group. However, no correlations were detected between NUCB2/nesfatin-1 and depression (PHQ-9 score; *r* = -0.12, *p* = 0.52; Fig 1C), perceived stress (PSQ-20 total score; *r* = 0.05, *p* = 0.75; Fig 1D) as well as all PSQ-20-subscales (Table 2).

### Circulating NUCB2/nesfatin-1 levels are not associated with eating disorder symptoms in anorexic patients

**Fig 3:**
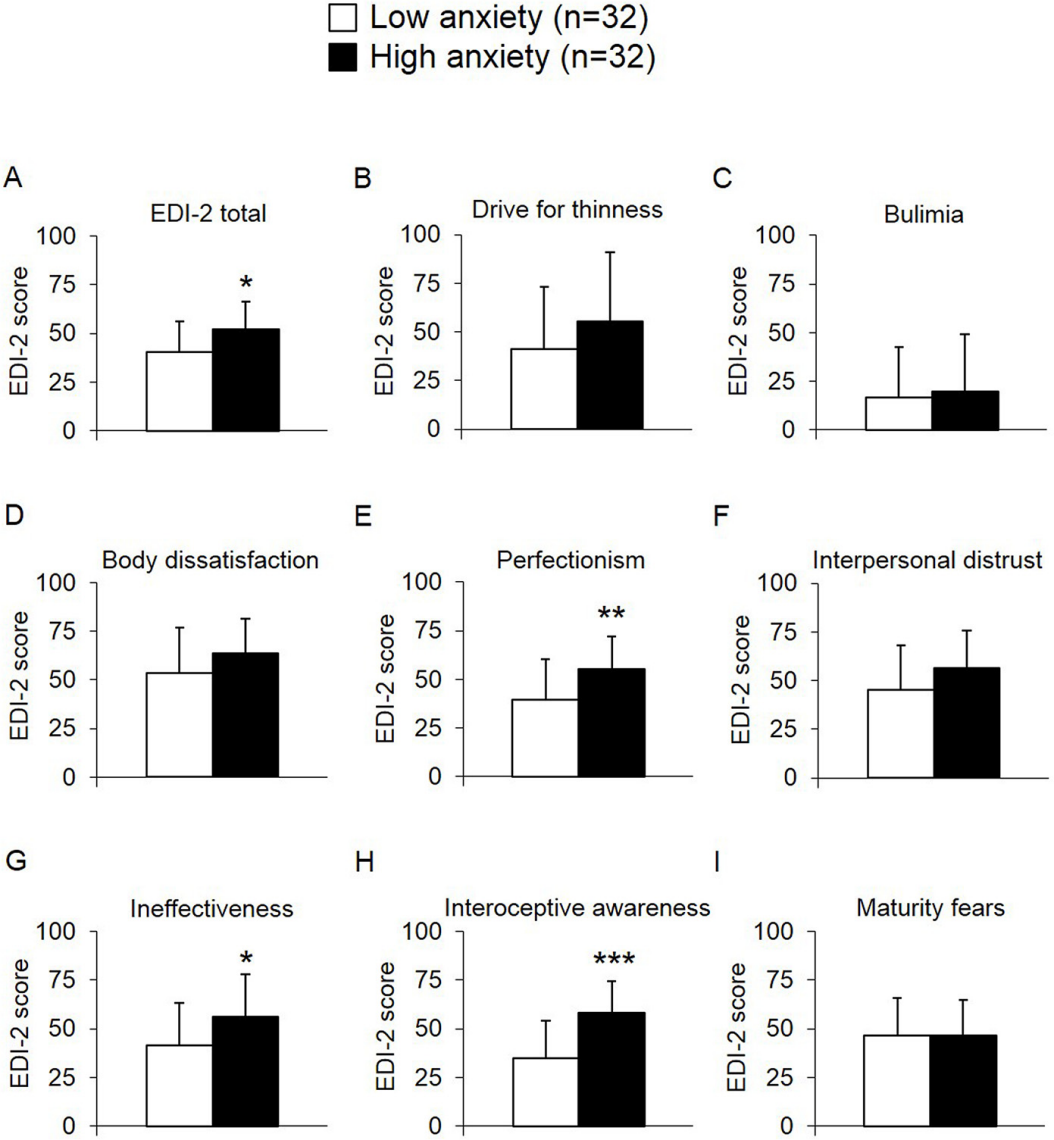
EDI-2 scores of anorexic patients with low *versus* high anxiety. Patients with high anxiety scores displayed a higher EDI-2 total score (A) and higher values of the subscales “perfectionism” (E), “ineffectiveness” (G) and “interoceptive awareness” (H) compared to those with low anxiety, while the subscales “drive for thinness” (B), “bulimia” (C), “body dissatisfaction” (D), “interpersonal distrust” (F) and “maturity fears” (I) did not differ between the two groups. Differences between groups were calculated using t-tests. Data are expressed as mean ± standard deviation. * *p* < 0.05, ** *p* < 0.01 and *** *p* < 0.001. Abbreviations: EDI-2, eating disorder inventory.

Patients with high anxiety levels exhibited higher scores on the EDI-2 total score compared to the low anxiety group (+30%, *p* = 0.01; Fig 3A). This resulted from their higher scores on the EDI-2 subscales “perfectionism” (+40%, *p* = 0.009; Fig 3E), “ineffectiveness” (+36%, *p* = 0.03; Fig 3G) and “interoceptive awareness” (+68%, *p* < 0.001; Fig 3H), while no significant differences were observed for the subscales “drive for thinness” (*p* = 0.18; Fig 3B), “bulimia” (*p* = 0.70; Fig 3C), “body dissatisfaction” (*p* = 0.12; Fig 3D), “interpersonal distrust” (*p* = 0.09; Fig 3F), and “maturity fears” (*p* = 0.96; Fig 3I). However, no correlations were detected between circulating NUCB2/nesfatin-1 levels and the EDI-2 total score (*r* = -0.14, *p* = 0.51; Fig 1E) or all subscale scores in the population of anorexic patients (Table 2).

### Circulating NUCB2/nesfatin-1 levels tend to be higher in normal weight compared to anorexic patients and to correlate positively with anxiety, depression, perceived stress and eating disorder symptoms in normal weight patients

In a small sample we compared circulating NUCB2/nesfatin-1 levels of 10 anorexia nervosa patients with a sample of 10 normal weight subjects matched for sex, age and anxiety scores. The socioeconomic characteristics of the anorexia nervosa and the normal weight patient populations are described in S2 Table. The main and secondary diagnoses and the duration of disease in matched normal weight and anorexia nervosa patients are shown in S3 Table.

Normal weight patients displayed about twofold higher NUCB2/nesfatin-1 levels compared to matched anorexia nervosa patients (0.393 ± 0.368 *vs*. 0.156 ± 0.09 ng/ml; Fig A in S1 File). However, this result only tended to be significant (*p* = 0.08). In the normal weight patient subgroup, NUCB2/nesfatin-1 also showed the tendency towards a correlation with GAD-7 scores, but also missed significance (*r* = 0.64; *p* = 0.06; Fig B in S1 File). Strong positive correlations were observed for NUCB2/nesfatin-1 and PHQ-9 (*r* = 0.70; *p* = 0.03; Fig C in S1 File), PSQ-20 (*r* = 0.78; *p* < 0.01; Fig D in S1 File), and EDI-2 (*r* = 0.79; *p* = 0.03; Fig E in S1 File) scores.

## Discussion

Nesfatin-1 has been implicated in the regulation of satiety and body weight [44]. Early on, the involvement of NUCB2/nesfatin-1 was also shown in the stress response of rodents and more recently also an implication of NUCB2/nesfatin-1 in the regulation of anxiety and depression in humans was described [20]. In the present study we evaluated the association between circulating NUCB2/nesfatin-1 and anxiety in anorexia nervosa. As hypothesized, we observed significantly higher NUCB2/nesfatin-1 plasma levels in female anorexic patients displaying high anxiety scores compared with those exhibiting low anxiety scores which resulted in a positive correlation of NUCB2/nesfatin-1 with anxiety scores. However, no correlations were detected for NUCB2/nesfatin-1 with perceived stress, depression and eating disorder-related behaviors, attitudes and cognitions.

The result of a positive association of NUCB2/nesfatin-1 with anxiety is in line with our previous finding in female obese patients showing higher levels of NUCB2/nesfatin-1 in subjects with increased anxiety which was also reflected in a strong correlation of NUCB2/nesfatin-1 with anxiety scores [19]. In addition, these data corroborate preclinical findings of a link between nesfatin-1 and anxiety as well as stress-induced anxiety-like behavior in rodents [21–24]. Interestingly, one recent human study detected decreased NUCB2/nesfatin-1 plasma levels in male patients with the diagnosis of a generalized anxiety disorder compared to healthy controls [26] which could point towards a sex-specific regulation of NUCB2/nesfatin-1. This concept was also proposed in a study investigating brain NUCB2 expression in depressed suicide victims compared to controls who did not die as a consequence of neuropsychiatric disorders. While male subjects had higher midbrain NUCB2 mRNA levels compared to controls, in females an inverse relationship was described [6]. This would suggest an inverse expression of central and peripheral NUCB2/nesfatin-1 in males and females under conditions of anxiety and depression which warrants further research to investigate this possible sex-specific effect.

In light of the proximity of the psychological constructs of depression and anxiety we also expected a positive association of NUCB2/nesfatin-1 and depression in the present study population. One study reported a positive correlation of plasma NUCB2/nesfatin-1 with depression (measured by Hamilton Depression Rating Scale, HAM-D) in a mixed-sex population of patients diagnosed with major depressive disorder and a healthy control group [25]. In addition, we recently described a positive association of NUCB2/nesfatin-1 with depression (measured by PHQ-9) in obese women [19]. However, in the present population of female subjects with anorexia nervosa we did not observe a correlation of NUCB2/nesfatin-1 with depression as indicated by PHQ-9. Moreover, an association of circulating NUCB2/nesfatin-1 with perceived stress, a finding reported before in obese women [19], was also absent in the current study population. Besides the fact that the above mentioned sex-specific effects might have contributed to these differential results, the psychometric mean of assessment of depression could also influence the observed outcome. Although HAM-D and PHQ-9 both measure and quantify depression, they may determine different aspects since the HAM-D is an external while the PHQ-9 is a self-assessment questionnaire [45, 46]. However, most likely these differential results in obese *versus* anorexic patients are due to a stronger association of NUCB2/nesfatin-1 with anxiety than depression or perceived stress that emerges when NUCB2/nesfatin-1 plasma levels are overall low as observed in the present anorexic population (0.22 ± 0.19 ng/ml) compared to obese women (0.40 ± 0.13 ng/ml) [19].

Nesfatin-1 is a well-established anorexigenic hormone in rodents and NUCB2/nesfatin-1 blood levels were reported to be reduced in patients with restricting-type anorexia nervosa [13] suggesting an implication of this peptide in the onset, course or maintenance of the disease. However, no further studies in anorexic subjects were conducted to date. Moreover, until now studies are lacking that investigate the relationship between NUCB2/nesfatin-1 and behaviors, cognitions or attitudes in eating disorders. In the present study, we did not detect an association of circulating NUCB2/nesfatin-1 with the total score of EDI-2 or its subscales in subjects with anorexia nervosa. Thus, NUCB2/nesfatin-1 does not seem to be involved in the specifically pathological eating behavior displayed by anorexic patients. However, in light of the correlation of NUCB2/nesfatin-1 with anxiety one might hypothesize that NUCB2/nesfatin-1 is primarily involved in the mediation of anxiety symptoms and subsequently influences food intake, potentially contributing to worse outcomes in a very anxious subgroup of anorexia nervosa patients.

Early on, a positive association of circulating NUCB2/nesfatin-1 and BMI has been reported in normal weight to obese [7] and normal weight study populations [14]. In line with these data, anorexic subjects were reported in one study to have lower NUCB2/nesfatin-1 levels compared to healthy controls [13]. However, in the present study we did not observe an association of NUCB2/nesfatin-1 with BMI in anorexic patients. This is most likely due to the low BMI spectrum investigated ranging from 9 to 19 kg/m^2^ and overall very low NUCB2/nesfatin-1 plasma levels. Interestingly, also recent studies challenged the initially described positive association of NUCB2/nesfatin-1 and BMI by reporting a negative correlation in normal weight to low grade obese subjects [15–18], or a lacking correlation in a very obese population [19]. In light of the emerging evidence on the association between NUCB2/nesfatin-1 and anxiety these inconsistent findings might be likely due to confounding factors such as anxiety which have not been assessed in these studies.

In the small sample of normal weight patients NUBC2/nesfatin-1 showed a tendency towards a correlation with anxiety scores, however, without reaching statistical significance. These significances were reached with positive correlations between NUCB2/nesfatin-1 and depression, perceived stress and eating disorder symptoms giving rise to a regulation of NUCB2/nesfatin-1 in a similar manner compared to anorexia nervosa. Normal weight patients also displayed a tendency towards a significant difference with markedly higher NUCB2/nesfatin-1 levels compared to anorexic patients which may be due to the influence of the higher body weight compared to anorexia nervosa. The narrowly missed significances may be due to the small group size. Future studies with larger sample sizes are warranted to investigate possible differences in the regulation of NUCB2/nesfatin-1 with regards to anxiety, depression, perceived stress, and eating disorder symptoms in normal weight patients.

Several limitations have to be considered when interpreting these data. First, we did not investigate NUCB2/nesfatin-1 in its association with nosological diagnoses as generalized anxiety disorder or major depressive disorder and did not provide a standardized diagnostic assessment. However, one has to take into account that most likely it is not the diagnostic classification but rather the individually perceived burden of symptoms as anxiousness, depressiveness, perceived stress or eating disorder symptoms such as “body dissatisfaction” or “ineffectiveness that might be associated with altered hormone levels. In this context, constructs like anxiousness or depressiveness most likely are better operationalized as continuous rather than categorial variables. Nevertheless, as mentioned above the psychometric instruments used in this study are self-assessment questionnaires which might be affected by inaccurate self-reporting caused by recall bias, social desirability or difficulties in self-observation. However, these questionnaires are well validated and suitable for bedside application for the measurement of perceived burden of symptoms in naturalistic study designs. Second, we did not employ a healthy control group. However, it was not our main aim to compare NUCB2/nesfatin-1 levels of healthy controls with anorexic subjects but to investigate whether NUCB2/nesfatin-1 shows a positive correlation with anxiety also in female anorexic subjects. Third, due to the rather small sample sizes we potentially were not able to detect associations between peripheral NUCB2/nesfatin-1 levels and scores of PHQ-9, PSQ-20 and EDI-2. This warrants further examination in future studies. Finally, circulating NUCB2/nesfatin-1 levels might be affected by circadian variations and all variables studied in the present investigation are known to be affected by several other influencing factors. However, considering all relevant confounding variables would require larger study populations and a measurement of a broad variety of items which is beyond the scope of the current first study. This should be addressed in future studies using multiple regression analysis models.

## Conclusions

As the main finding supporting the initial hypothesis we observed a significant correlation of NUCB2/nesfatin-1 with anxiety in a population of anorexic patients. Unexpectedly, no association was found for NUCB2/nesfatin-1 with depression and perceived stress, giving rise to a predominant link of NUCB2/nesfatin-1 with anxiety and anxiety-like behaviors. Longitudinal and interventional studies in different populations are needed in order to further investigate NUCB2/nesfatin-1’s implication in the regulation of anxiety and the causal direction of this interrelation. In addition, we exploratively did not observe an association of NUCB2/nesfatin-1 with eating disorder symptoms as measured by the EDI-2 making NUCB2/nesfatin-1 unlikely to be directly involved in eating disorder pathology in anorexia nervosa.

In synopsis with the existing literature we therefore hypothesize that NUCB2/nesfatin-1 might be primarily involved in the mediation of anxiety and that its well-described influence on food intake and body weight is independent from disordered eating – at least in patients with anorexia nervosa. However, due to the anorexigenic properties of NUCB2/nesfatin-1 more anxious anorexic patients might be at greater risk for unfavorable outcomes than those with lower tendency to anxiousness.

## Supporting Information

S1 File GAD-7, PHQ-9, PSQ-20 and EDI-2 scores and plasma NUCB2/nesfatin-1 levels in normal weight patients. Normal weight patients tended to display higher NUCB2/nesfatin-1 levels compared to anorexic patients (Fig A). Normal weight patients showed a positive correlation of NUCB2/nesfatin-1 with anxiety scores (Fig B), depression scores (Fig C), perceived stress scores (Fig D) and eating disorder symptoms (Fig E). Differences between groups were calculated using the t-test. Data are expressed as mean ± standard deviation. Distribution of the data was determined by the Kolmogorov-Smirnov test. Correlations were determined by Pearson’s or Spearman’s analyses depending on the distribution of the data. Values for *r* and *p* are indicated in the graph. Abbreviations: EDI-2, eating disorder inventory; GAD-7, general anxiety disorder questionnaire; NUCB2, nucleobindin2; PHQ-9, patient health questionnaire depression; PSQ-20, perceived stress questionnaire.
(PDF)

S1 Table Comorbidities and additional laboratory analyses in anorexia nervosa patients. (PDF)

S2 Table Demographic and socioeconomic characteristics of the matched normal weight and anorexia nervosa patients. (PDF)

S3 Table Main diagnoses of normal weight patients, duration of disease and comorbidities of matched normal weight and anorexia nervosa patients. (PDF)
