# Supplementary figures and images for: NUCB2/nesfatin-1 Is Associated with Elevated Levels of Anxiety in Anorexia Nervosa
Source: PLoS One. 2015 Jul 10;10(7):e0132058. doi: 10.1371/journal.pone.0132058 (PMC4498697; doi:10.1371/journal.pone.0132058)

□ Normal weight (n=10)  
■ Anorexia nervosa (n=10)

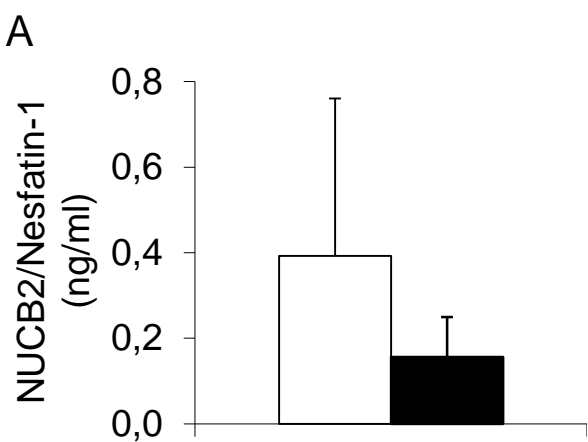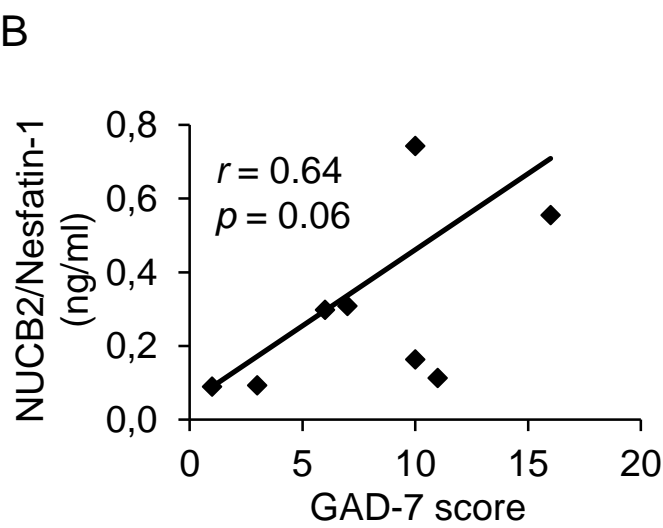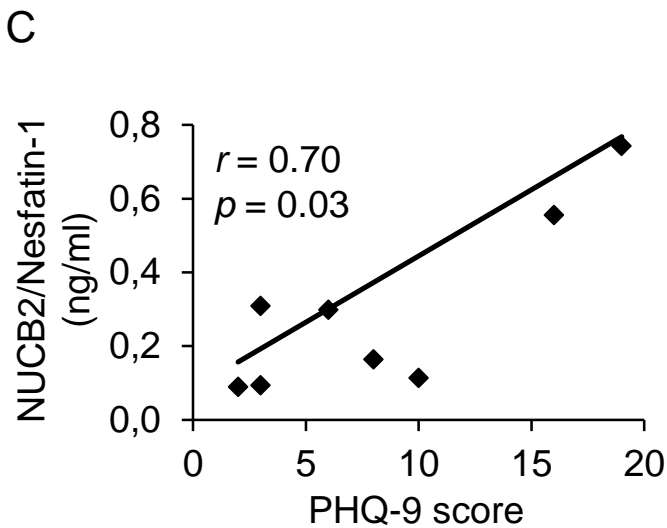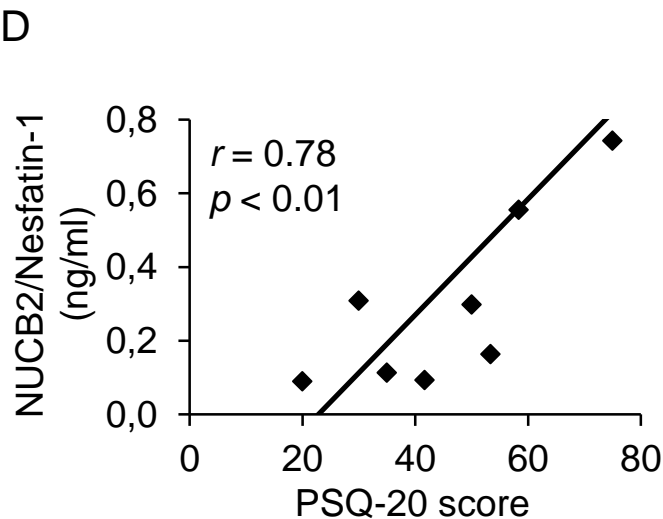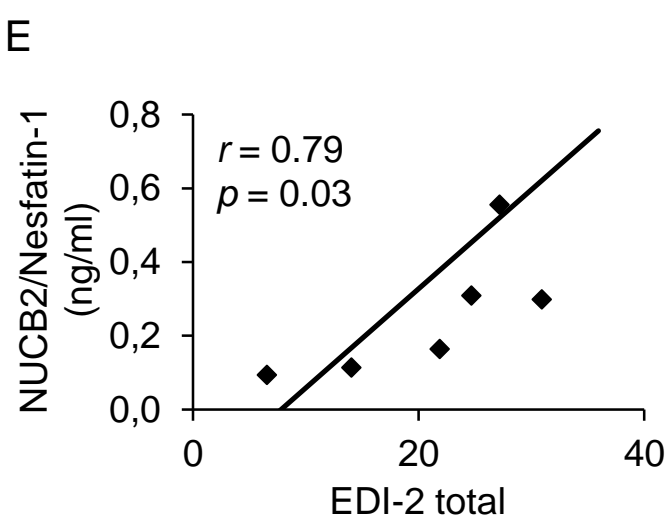

Supplement: S1 File — Normal weight patients tended to display higher NUCB2/nesfatin-1 levels compared to anorexic patients (Fig A). Normal weight patients showed a positive correlation of NUCB2/nesfatin-1 with anxiety scores (Fig B), depression scores (Fig C), perceived stress scores (Fig D) and eating disorder symptoms (Fig E). Differences between groups were calculated using the t-test. Data are expressed as mean ± standard deviation. Distribution of the data was determined by the Kolmogorov-Smirnov test. Correlations were determined by Pearson’s or Spearman’s analyses depending on the distribution of the data. Values for r and p are indicated in the graph. Abbreviations: EDI-2, eating disorder inventory; GAD-7, general anxiety disorder questionnaire; NUCB2, nucleobindin2; PHQ-9, patient health questionnaire depression; PSQ-20, perceived stress questionnaire. (PDF) [file pone.0132058.s001.pdf]
