# Supplementary material for: NUCB2/nesfatin-1 Is Associated with Elevated Levels of Anxiety in Anorexia Nervosa
Source: PLoS One. 2015 Jul 10;10(7):e0132058. doi: 10.1371/journal.pone.0132058 (PMC4498697; doi:10.1371/journal.pone.0132058)
Supplement: S1 Table — (PDF) [file pone.0132058.s002.pdf]

S1 Table. Comorbidities and additional laboratory analyses in anorexia nervosa patients.

**Parameter**	**Low anxiety (n=32)**	**High anxiety (n=32)**	**Missing data**	***p***
Psychiatric/psychological comorbidities			0	
Alcohol abuse	0	2		n.c.
Major depressive disorder	5	7		n.c.
Adjustment disorder	2	1		n.c.
Somatoform disorder	3	0		n.c.
Panic disorder	1	0		n.c.
Obsessive-compulsive disorder	1	3		n.c.
Personality disorder/accentuation	1	2		n.c.
Somatic Comorbidities			0	
Hypothyreosis	0	2		n.c.
Epilepsy	1	1		n.c.
Ascites	0	3		n.c.
Pericardial effusion	3	5		n.c.
Chronic heart failure	1	0		n.c.
Interstitial nephritis	1	0		n.c.
Urinary stasis	0	1		n.c.
Gastritis	3	0		n.c.
Bronchial asthma	1	0		n.c.
Sinusitis	0	1		n.c.
Locomotor system disorders	1	0		n.c.
Osteopenia	1	1		n.c.

Additional laboratory analyses				
TSH (mU/l)	1.7 (1.3/3.0)	2.6 (2.0/3.4)	2	**0.03**[a]
CRP (mg/l)	0.4 (0.2/0.9)	0.2 (0.2/0.7)	2	0.32[a]
Glucose (mg/dl)	74.6 ± 10.4	70.0 ±12.1	1	0.12[b]
Cortisol (nmol/l)	866.4 ± 363.1	845.9 ± 364.4	5	0.83[b]

Statistical analyses: Normal distribution was determined by Kolmogorov-Smirnov test. Differences between groups: [a] Mann-Whitney-U-test, data expressed as median (25. percentile/75. percentile); [b] t-test, data are expressed as mean ± standard deviation. Significant differences are displayed in bold. Abbreviation: n.a., not applicable; n.c., not calculated.
