# Supplementary material for: NUCB2/nesfatin-1 Is Associated with Elevated Levels of Anxiety in Anorexia Nervosa
Source: PLoS One. 2015 Jul 10;10(7):e0132058. doi: 10.1371/journal.pone.0132058 (PMC4498697; doi:10.1371/journal.pone.0132058)
Supplement: S2 Table — (PDF) [file pone.0132058.s003.pdf]

S2 Table. Demographic and socioeconomic characteristics of the matched normal weight and anorexia nervosa patients.

**Parameter**	**Normal weight patients (n=10)**	**Anorexia nervosa patients (n=10)**	**Missing data**	***p***
Demographic characteristics				
Age (years)	37.0 ± 11.9	36.4 ± 11.1	0	0.91
Body mass index (kg/m^2)	20.5 ± 1.5	14.0 ± 3.1	0	**<0.001**
Socioeconomic characteristics				
Living in a partnership (yes/no)	5/4	3/5	3	0.46
Level of education			3	0.31
university entrance diploma	4	1		
vocational diploma	1	0		
level 1 certificate	3	5		
certificate of secondary education	1	2		
without	0	0		
Current employment (yes/no)	7/2	3/5	3	0.09
Unemployment during past 5 years (yes/no)	1/8	1/7	3	0.93
Psychometric assessment				
GAD-7 total score	9.0 ± 4.8	11.5 ± 4.3	0	0.23
PHQ-9 total score	9.3 ± 6.0	14.4 ± 7.5	1	0.12
PSQ-20 total score	51.0 ± 19.6	69.5 ± 15.4	0	**0.03**
EDI-2 total score	23.9 ± 9.6	46.2 ± 9.4	4	**<0.001**

Statistical analyses: Normal distribution was determined by Kolmogorov-Smirnov test. Differences between groups were determined by t-tests for demographic characteristics and psychometric scores and by χ^2-tests for socioeconomic characteristics. Data are expressed as mean ± standard deviation. Significant differences are displayed in bold. Abbreviations: EDI-2, eating disorder inventory; GAD-7, general anxiety disorder questionnaire; PHQ-9, patient health questionnaire depression; PSQ-20, perceived stress questionnaire.
