# Supplementary material for: NUCB2/nesfatin-1 Is Associated with Elevated Levels of Anxiety in Anorexia Nervosa
Source: PLoS One. 2015 Jul 10;10(7):e0132058. doi: 10.1371/journal.pone.0132058 (PMC4498697; doi:10.1371/journal.pone.0132058)
Supplement: S3 Table — (PDF) [file pone.0132058.s004.pdf]

S3 Table. Main and secondary diagnoses, duration of disease and additional laboratory analyses of matched normal weight and anorexia nervosa patients.

Parameter	Normal weight patients (n=10)	Anorexia nervosa patients (n=10)	Missing data	*p*
Main diagnoses			0	
Adjustment disorder (ICD-10 F43.2)	2	n.a.		
Somatoform pain disorder (ICD-10 F45.4)	6	n.a.		
Generalized anxiety disorder (ICD-10 F41.1)	2	n.a.		
Anorexia nervosa subtype (ICD-10 F50.0)				
restricting type	n.a.	7		
purging type	n.a.	1		
atypical anorexia nervosa	n.a.	2		
Secondary diagnoses (psychiatric/psychological)				
Major depressive disorder	3	2		n.c.
Adjustment disorder	0	2		n.c.
Somatoform disorder	2	0		n.c.
Generalized anxiety disorder	1	0		n.c.
Secondary diagnoses (somatic)				
Panic disorder	0	1		n.c.
Tinnitus	1	0		n.c.
Liver hemangioma	1	0		n.c.
Substance abuse	2	0		n.c.
Autoimmune thyreoditis	1	0		n.c.
Hypothyreosis	0	1		n.c.
Interstitial nephritis	0	1		n.c.
Locomotor system disorders	2	0		n.c.
Osteopenia	0	1		n.c.

Duration of disease			2	
< ½ year	1	0		n.c.
½ to 1 year	1	0		n.c.
1-2 years	2	2		n.c.
2-5 years	4	2		n.c.
5-10 years	1	1		n.c.
10-20	0	1		n.c.
> 20 years	0	3		n.c.
Additional laboratory analyses				
TSH (mU/l)	1.9 (1.4/2.5)	2.0 (0.9/3.1)	0	0.94[a]
CRP (mg/l)	0.4 (0.3/1.4)	0.3 (0.2/0.5)	1	0.27[a]
Glucose (mg/dl)	82.2 ± 6.6	72.4 ± 9.7	0	**0.02[b]**
Cortisol (nmol/l)	553.4 ± 260.1	867.2 ± 352.1	1	**<0.05**[b]

Statistical analyses: Normal distribution was determined by Kolmogorov-Smirnov test. Differences between groups: [a] Mann-Whitney-U-test, data expressed as median (25. percentile/75. percentile); [b] t-test, data expressed as mean ± standard deviation. Significant results are displayed in bold. Abbreviation: CRP, c-reactive peptide; n.a., not applicable; n.c., not calculated; TSH, thyroid stimulating hormone.
